# Laboratory Scale Evaluation of Fertiliser Factory Wastewater Treatment through Membrane Distillation and Reverse Osmosis

**DOI:** 10.3390/membranes11080610

**Published:** 2021-08-10

**Authors:** M. Tagliabue, J. Tonziello, A. Bottino, G. Capannelli, A. Comite, M. Pagliero, F. Boero, C. Cattaneo

**Affiliations:** 1 Renewable Energy and Environmental Laboratories, Eni S.p.A., F. Maritano 26, I-20097 San Donato Milanese, Italy; Marco.Tagliabue@eni.com (M.T.); Jacopo.Tonziello@eni.com (J.T.); 2 Department of Chemistry and Industrial Chemistry, University of Genoa, Dodecaneso 31, I-16146 Genoa, Italy; bottino@chimica.unige.it (A.B.); gustavo.capannelli@unige.it (G.C.); marcello.pagliero@unige.it (M.P.); 3 TICASS S.c.r.l., B. Bosco 57/4, I-16121 Genoa, Italy; federica.boero@ticass.it (F.B.); claudia.cattaneo@ticass.it (C.C.)

**Keywords:** fertiliser wastewater, ammonium, phosphates, vacuum membrane distillation, reverse osmosis

## Abstract

The incumbent water stress scenario imposes wastewater valorisation to freshwater, promoting technology for its effective treatment. Wastewater from fertiliser factories is quite problematic because of its relevant acidity and solute content. Its treatment through vacuum membrane distillation (VMD) was evaluated through laboratory scale tests at 40 °C and 25 mbar vacuum pressure with polytetrafluoroethylene and polypropylene flat-sheet porous membranes. The wastewater from a partially disused Italian industrial site was considered. VMD distillate fluxes between 22 and 57.4 L m^−2^ h^−1^ (LMH), depending on the pore size of the membranes, along with very high retention (R > 99%) for anions (Cl^−^, NO_3_^−^, SO_4_^2−^, PO_4_^3−^), NH_4_^+^, and chemical oxygen demand (COD) were observed. Laboratory scale reverse osmosis (RO) tests at 25 °C and increasing of the operating pressure (from 20 bar to 40 bar) were carried out with a seawater desalination membrane for comparison purposes. Permeability values around 1.1 LMH/bar almost independently of the operating pressure were observed. Lower retentions than those measured from VMD tests were found. Finally, for any given RO operating pressure, the flux recovery ratio (FRR) calculated from permeate fluxes measured with pure water before and after wastewater treatment was always much lower that evaluated for VMD membranes.

## 1. Introduction

Europe is not perceived as a dry continent. In fact, water stress affects several European countries, mainly in the Mediterranean region. This is because of both intensive resource exploitation and adverse climate change [1]. Within this scenario, even wastewater has to be considered a potential freshwater resource, promoting technology for its effective treatment. Industry must take part in this challenge: Eni Rewind (former, Syndial, Eni subsidiary for environmental activity) treated 20 Mm^3^ of industrial wastewater from Italian sites in 2013, and this volume is continuously rising [2].

Fertiliser factories wastewater (FFWW) is quite problematic owing its relevant acidity and solute content [3]. The specific chemical characteristics of fertilizer wastewater depend on the particular production processes (e.g., urea, diammonium or monoammonium phosphate, potash). Nitrogen and phosphorus compounds are specific concerns owing to eutrophication risk. FFWWs are usually treated by means of traditional physical-chemical and biological methods. Coagulation was applied to superphosphate removal [4] and the use of hydrated lime to precipitate insoluble calcium-based salts was shown, which can be reused in the fertiliser production process [5,6]. Ion exchange with anion and cation exchange resins can effectively be used to remove nitrogen as ammonium and nitrate when their concentration is low (e.g., <180 mg/L for NH_4_^+^) [7]. Interesting results were also obtained using a natural zeolite, namely clinoptilolite, as a cation exchanger [8]. In any case, adsorption and ion exchange seem more suitable as a refining process of the treated wastewater. Bhandari et al. [9] studied the application of hydrodynamic cavitation, showing a comparable nitrogen removal efficiency to adsorption. The conventional biological nitrogen and phosphorus removal treatments are widely applied to several types of industrial wastewater [10]. Phosphate rich wastewaters (about 4.5 g/L) were pretreated with lime and then by a two-stage biological process, achieving a P removal efficiency of about 92% [11]. One of the main hurdles of FWW is related to the high salinity and chloride concentration, which create a harsh environment for bacteria. Ucisiz and Henze [12] showed that a denitrifying biomass can work with up to 96 g/L of chloride, but with removal rates tenfold less than in municipal wastewater. Pam and Bui successfully employed microalgae to treat a weak fertilizer wastewater with neutral pH and low nitrogen and phosphorus concentrations [13]. Yavari et al. [14] proposed the use of teak tree for the phytoremediation of nitrogen rich fertilizer factory wastewater to minimize the effects of nitrogen reintroduction in the environment owing to the short life cycle of common wetland plants. The N removal efficiency was about 77% over a period of 2 months and, although the proposed solution would be very interesting, it needs further studies before its application to a large volume of wastewater. Exergy analysis was applied to evaluate the integration of some physicochemical and biological processes to treat fertilizer wastewater [15]. Finally, Singh et al. [16] suggested the catalytic peroxidation treatment for fertilizer wastewater, where the nitrogen and phosphorus compounds cannot be easily removed by physicochemical and biological methods.

Surprisingly, scant attention has till now been paid to the use of membrane processes that, for many years, have found well established applications for the recovery of high quality water from a large variety of wastewaters [17,18,19]. In fact, to the best of our knowledge, only two papers based on the application of pressure-driven membrane processes, such as reverse osmosis (RO) and nanofiltration (NF), have been reported in the scientific literature [20,21]. In the first of these two papers, Karabelas et al. [20] used four different RO membranes for bench and short-term pilot scale tests with two types of streams: one rich in nitrate and ammonium ions, and another highly acidic and rich in fluorosilicic and fluoride ions. In the second and more recent paper, Dolar et al. [21] performed laboratory scale tests on artificial model solutions and on real wastewater to investigate the removal efficiency of six different membranes (three NF and three RO) to reduce phosphate and fluoride.

State-of-the-art treatments operated by Eni Rewind entail at least liming as the first step and reverse osmosis (RO) as the last one, once a large part of solutes has been removed to lower the osmotic pressure. Presently, 200 m^3^/day of wastewater from a partially disused Italian industrial site is managed while obtaining freshwater suitable for surface discharge. Liming sludge is dewatered and landfilled, while RO concentrate goes through underground injection. Both landfill and injection wells are in the same site.

Membrane distillation (MD) is a new and emerging thermally driven process and, since the 1990s, more than 50 reviews can be found from Web of Science using the keywords “membrane distillation review” for the title. The topic is in constant evolution and relevant reviews [22,23,24,25,26] and several interesting chapters and books have been published [27,28,29,30,31].

The working principle of MD is based on the vapour pressure difference between the opposite sides of porous hydrophobic membranes. The latter ones act as contactors, allowing distillate vapour flow from the compartment containing warm feed. The main configurations for membrane distillation are as follows:

- vacuum membrane distillation (VMD), where the volatile components are removed from one of the membrane surfaces by vacuum;
- sweep gas membrane distillation (SGMD), where the volatile components are removed by flowing a sweep gas on one of the membrane surfaces;
- air-gap membrane distillation (AGMD), where on the vapour side of the membrane a cold surface recovers the condensate;
- direct contact membrane distillation (DCMD), where a colder liquid is fed to the membrane side where the vapor and volatile component are transferred.

Distillate vapour can be condensed into liquid freshwater, while concentrate remains as liquid waste. MD provides water of better quality than RO, as non-volatile solutes are, in theory, completely retained by the membrane. A potentially reduced tendency to fouling [32,33] and much lower polarisation [34,35] are claimed as benefits of MD with respect to RO. SGMD, VMD, and DCMD were investigated to remove volatile ammonia together with water vapour [36,37,38,39]. Using this approach, the pH of the feed solution needs to be raised to more than neutrality to improve the ammonia removal efficiency into the permeate. In the specific case of DCMD, an acidic aqueous phase is fed on side of the membrane to improve the removal effectiveness owing to the acid base reaction, which increases the driving force for ammonia mass transfer. A similar approach was used for some wastewater treatment by MD. Fida et al., used MD as a post-treatment for nitrogen removal from anaerobic fluidized bed membrane bioreactor [40]. Nevertheless, with real wastewater, the correction of pH at higher values can induce precipitation and cleaning procedures are required to keep under control the fouling phenomena and the process performance [41]. Another approach of wastewater treatment uses a low pH during MD to limit the ammonia transfer and the main role of MD was to concentrate or dewater the nitrogen-rich wastewater [42,43].

The present paper reports the results of a preliminary study aimed at verifying the feasibility of treating FFWW with membrane distillation. Owing to the acidic nature of the FFWW, MD was used to concentrate the wastewater and to recover a good permeate water. Vacuum membrane distillation (VMD) tests were carried out with some types of porous hydrophobic polymeric membranes with different porosity and permeability. Owing to variability of the FWW depending on its origin and on the limited number of papers dealing with the application of membrane processes on FWW, it became difficult to properly compare MD and RO technologies on only the literature findings. Therefore, within the aim of the present investigation, VMD results were then compared to those of laboratory scale tests obtained with an RO membrane.

## 2. Materials and Methods

### 2.1. Membranes

Flat-sheet polytetrafluoroethylene (PTFE) and polypropylene (PP) membranes for VMD tests were provided by Membrane Solutions, LLC (USA) and Membrana GmbH (Germany), respectively. Flat sheets of polyamide thin film composite membranes for RO tests were cut from ADHR^TM^ spiral wound elements provided by GE Water & Process Technologies (USA). PP Accurel^TM^ and PTFE MS membranes are widely used in various studies in the field of membrane distillation. It was thus decided to select this type of membrane to study their behavior in this new application. The GE ADHR membrane is a common RO membrane suitable to preconcentrate water from any source and reduce wastewater volumes by increasing water reuse.

Relevant properties of different types of membrane supplied from the manufacturers are listed in Table 1 and Table 2. Detailed information on the structure and morphology of MD membranes was obtained from observations of membrane surfaces and cross section through a field emission scanning electron microscope (FESEM) Zeiss Supra 40VP^TM^.

### 2.2. Laboratory-Scale MD and RO Tests and Plants

**Figure 1:**
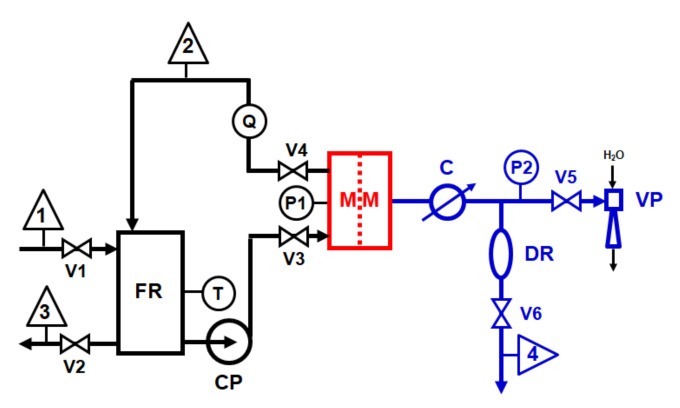
Laboratory-scale VMD plant scheme: 1 = feed inlet; 2 = concentrate outlet (recycled); 3 = feed outlet; 4 distillate outlet (recycled).

VMD tests were carried out through the laboratory-scale plant sketched in Figure 1. The membrane was housed in a rectangular cell (MM) with an effective membrane area of 22 cm^2^ and a channel height of 2 mm. The cell was fed at a constant flow rate of 500 L/h, measured through a variable area flow meter (Q) and pressure of 0.1 bar, measured by bourdon gauge (P1), through a magnetic centrifugal pump (CP) (mod. P051, Plastomec, IT; maximum capacity Q = 50 L/min, max head, H = 0.6 bar). Feed temperature of 40 °C was measured by a glass thermometer (T) immersed in a 2 L glass feed reservoir (FR) placed on a hot plate (not shown in Figure 1) with a proportional integrative derivative (PID) control. A water-jet vacuum pump (VP) was used for generating a pressure of 25 mbar measured through a vacuum gauge (P2). Distillate vapour was condensed through a glass condenser (C). A mixture of ethylene glycol and water kept at 0 °C was used as cooling fluid. Distillate flow rate was measured through the volume of distillate collected in a graduated glass cylinder (DR) after 10 min. All relevant parts of the feed recirculation loop were made in polypropylene (PP). A silicon rubber vacuum hose was used to connect the various vacuum line devices.

**Figure 2:**
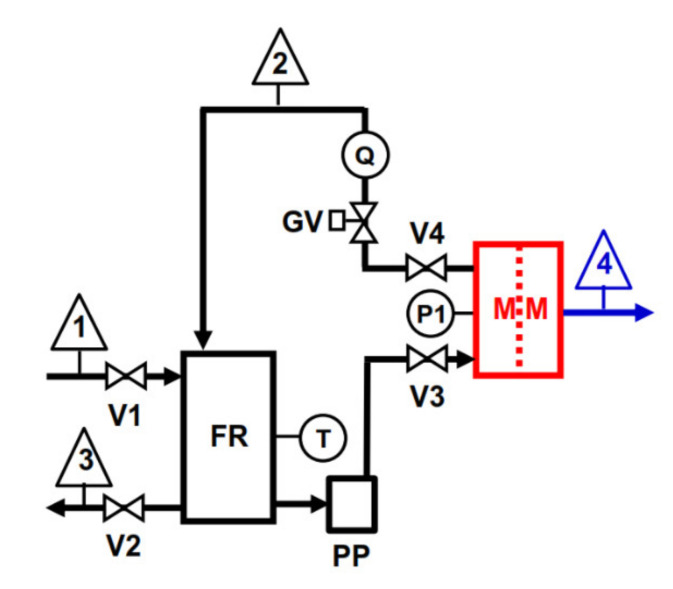
Laboratory-scale RO plant scheme: 1 = feed inlet; 2 = concentrate outlet (recycled); 3 = feed outlet; 4 = permeate outlet (recycled).

The scheme of the laboratory-scale plant used to perform RO tests (Figure 2) was similar to the one used for VMD tests shown in Figure 1. The piston pump (PP) (mod. 3CP1221, Catpumps, USA; capacity Q = 16 L/min, pressure range 6.9–138 bar) equipped with a variable-frequency-drive inverter speed control was used to deliver the feed solution to the rectangular flat cell (MM, effective membrane area of 66 cm^2^, and channel height of 2 mm) at a constant flow rate of 500 L/h, measured with the variable area flow meter (Q). A globe valve (GV) was employed to set the operating pressure (20, 30, and 40 bar), measured with the bourdon gauge (P). The feed temperature was measured with bimetal thermometer immersed in the 10 L feed reservoir and kept constant at 25 °C by a heat exchanger coil (not shown in Figure 2) located in the feed reservoir and connected to a thermostatic bath (not shown in Figure 2). All the relevant parts of the plant were made of stainless steel. Both VMD and RO tests were carried out by keeping the feed composition constant by recycling both VMD distillate (or RO permeate) and concentrate to the FR. VMD with wastewater lasted ca. 240 min, while the duration of RO for any of the above reported pressures was 60 min.

Pure water flux measurements (duration 60 min) were carried out before and after each type of wastewater test.

The relative flux reduction (RFR) was determined according to Equation (1):

*RFR* (%) = (1 − *J*_*ww*_ / *J*_*pw,i*_) ∗ 100 (1)

where *J_ww_*_and_ *J_pw,i_* are the fluxes measured with the wastewater and pure water before wastewater, respectively.

The flux recovery ratio (FRR) was calculated according to Equation (2):

*FRR* (%) = (*J*_*pw,f*_ / *J*_*pw,i*_) ∗ 100 (2)

where *J_pw,f_* is the pure water flux measured after wastewater.

VMD distillate and RO permeate quality were checked in term of pH, electrical conductivity (EC), anions (Cl^−^, F^−^, PO_4_^3−^, SO_4_^2−^) chemical oxygen demand (COD), and NH_4_^+^ levels measured on samples collected after 240 min for VMD and 60 min for RO. pH was measured through an Eutech PH510^TM^ pH-meter equipped with a Hanna HI1230^TM^ probe. EC was measured through a Hanna EC215^TM^ conductivity-meter equipped with a Hanna HI76303^TM^ probe. COD and NH_4_^+^ levels were determined through respective Merck Spectroquant^TM^ kits implemented on a Pharo 300^TM^ ultraviolet-visible spectrophotomer equipped with a TR320^TM^ reactor. Anions were evaluated with a Dionex DX120^TM^ ion chromatograph.

Membrane retention (R) was calculated according to Equation (3):

*R* (%) = (1 − *C_p_* / *C_f_*) ∗ 100 (3)

where *C_p_* and *C_f_* are the solute concentrations in the permeate and in the feed, respectively.

## 3. Results and Discussions

The main physical-chemical characteristics of the wastewater are shown in Table 3, together with Italian surface discharge limits [19]. Nitrogen and phosphorus are referred to as NH_4_^+^ and PO_4_^3−^, respectively, according to their actual speciation. As can be seen, contamination is mainly due to inorganics. In fact, COD is still within surface discharge limit. Moreover, the pH is quite low.

**Figure 3:**
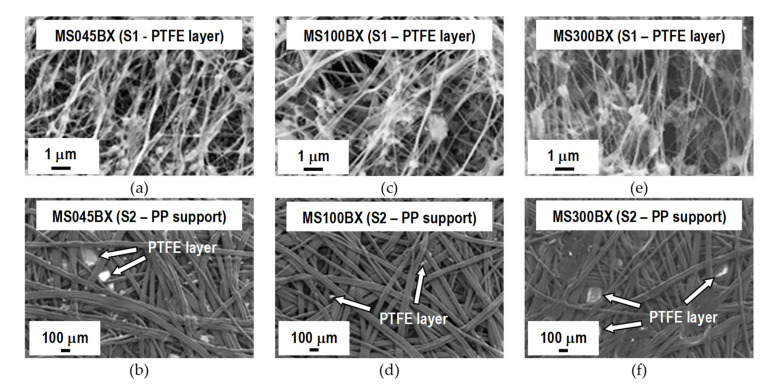
FESEM micrographs of the two surfaces (S1—PTFE layer and S2—PP support) of MS membranes. (**a**,**b**) MS045BX; (**c**,**d**) MS100BX; (**e**,**f**) MS300BX.

**Figure 4:**
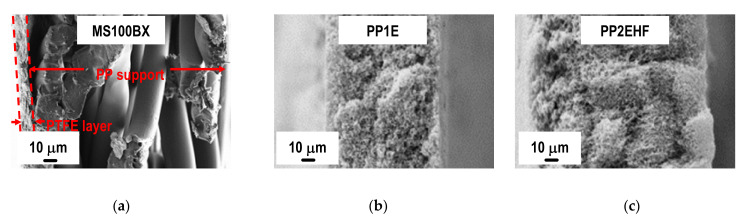
FESEM micrographs of the cross-section of the MS100BX (**a**), PP1E (**b**), and PP2EHF (**c**) membranes.

**Figure 5:**
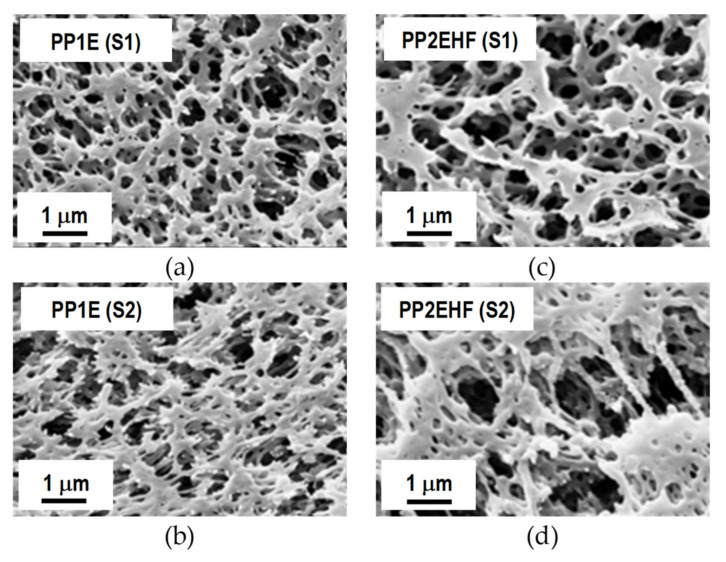
FESEM micrographs of the two surfaces (S1 and S2) of PP1E (**a**,**b**) and PP2EHF (**c**,**d**) membranes.

FESEM micrographs of the surfaces and cross section of the different types of VMD membrane are reported in Figure 3, Figure 4 and Figure 5. As can be seen from Figure 3, all the membrane solutions (MSs) membranes have similar texture. The PTFE layers show a microporous structure formed by nodes with irregular shape and size connected by fibrils of various lengths. Increasingly large cavities in agreement with the pore size values listed in Table 1 are clearly observed. The PP non-woven support is in all cases characterised by a highly macroporous structure formed by large fibers. The PTFE layer can be seen between some PP fibers. From the micrographs of the cross section of MS100BX^TM^ reported in Figure 4a, as a representative example for the three types of MS membranes, a remarkable difference between the thickness of the PTFE layer and that of the non-woven support is observed.

Further inspection of Figure 4 reveals a striking contrast between the structure and morphology of the cross section of the MS membrane with that of the two types of PP membranes supplied by Membrana. The porosity differences from one surface to the other of the latter membranes can be seen from the micrographs reported in Figure 5. It is apparent that the PP2EHF membrane has a larger pore size than the PP1E membrane with the presence of some bigger pores in the order of 2 µm. By applying image analysis using the open source software ImageJ, a mean pore size of about 0.24 µm and 0.63 µm was evaluated for the PP1E and the PP2EHF membranes, respectively. These findings are in good agreement with the isopropyl alcohol (IPA) bubble point and permeability values reported in Table 1.

**Figure 6:**
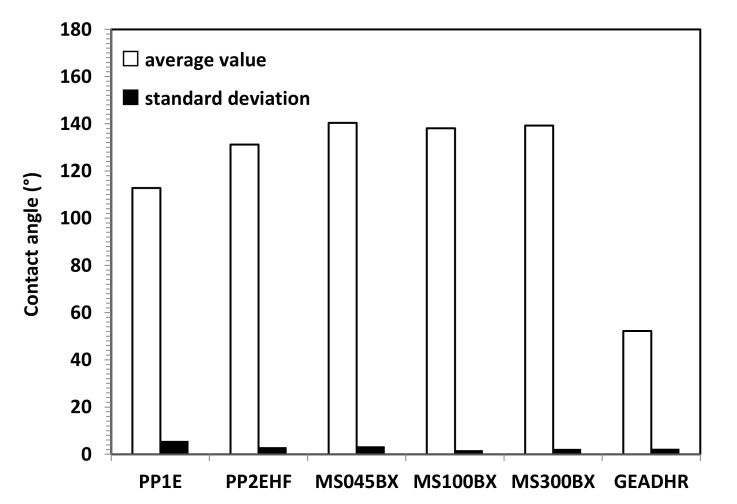
Average CA value and SD for VMD (PP and PTFE MS) and RO (GEADHR) membranes.

Figure 6 shows the water contact angle on the PP and PTFE MS membranes used in membrane distillation and the RO membrane. The membranes used for MD are clearly hydrophobic and PTFE MS membrane made of PTFE, exhibiting a contact angle approaching at 140°, higher than that of PP membranes. The PP2HF membrane exhibits a water contact angle higher than that of the PP1 membrane, probably owing to the higher surface porosity. The RO membrane shows a hydrophilic behavior with a water contact angle of about 45°, as expected because thin film composite membranes are usually made of a polymer as well as polyamide, which should allow a good solubility and diffusion of water.

**Figure 7:**
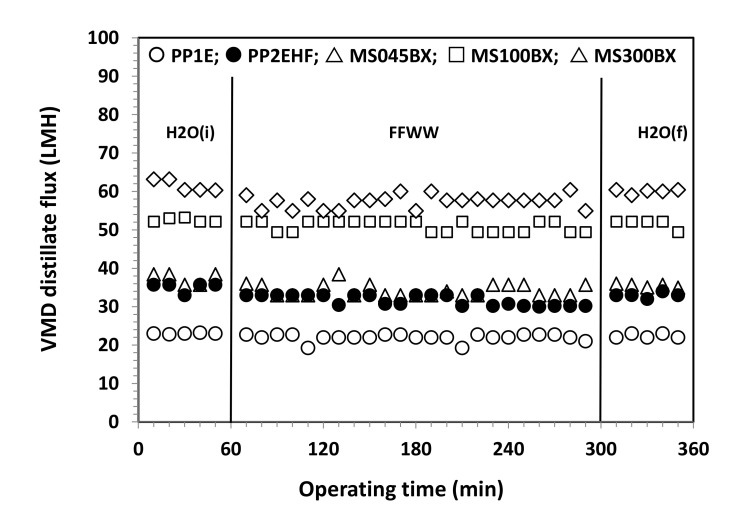
VMD distillate flux as a function of the operating time for PP and PTFE MS membranes.

**Figure 8:**
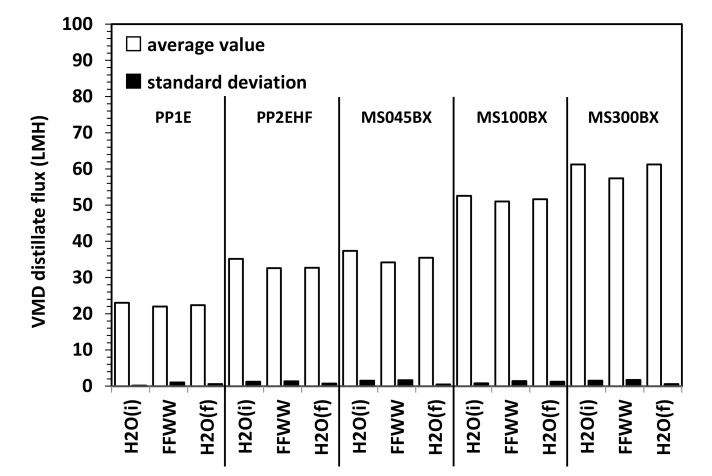
Average VMD distillate flux and SD for PP and PTFE MS membranes.

Figure 7 and Figure 8 refer to the results of VMD tests. As shown in Figure 7 for all membranes, only small fluctuations of the distillate flux (expressed in Lm^−2^ h^−1^ = LMH) connected to small changes of the temperature and vacuum pressure are observed. For the sake of a better comparison, average fluxes along with standard deviations (SD) are shown in Figure 8. The distillate flux with FFWW appears only slightly lower that than measured at the beginning of the VMD test with pure water, H_2_O (i). The distillate flux is higher for the thinner PTFE MS membranes and increases with the increasing pore size and decreasing bubble point (Table 1). For the same type of membranes, there is a good agreement between the evolution of the distillate flow and the porous structure of the selective layer observed by the FESEM images. The phenomenon is best seen in the case of PP Accurel^TM^ membranes, which have a less complex porous structure than that of MS membranes. For the latter ones, however, it can be observed how the MS100BX and MS300BX membranes with a very similar porous structure (despite the different nominal size of the pores) offer very close distillate flux. By repeating the pure water test, H_2_O (f), after wastewater treatment, an almost complete recovery of the initial pure water flux H_2_O (i) is obtained for all types of membranes.

**Figure 9:**
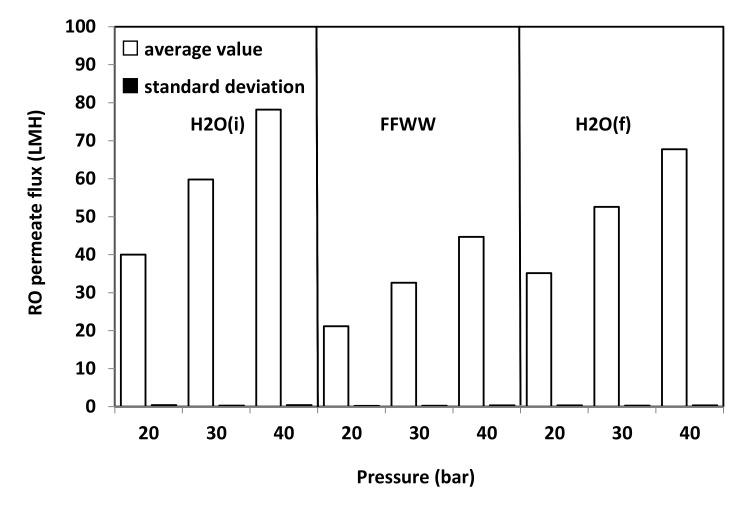
Average RO permeate flux and SD vs. operating pressure of the GEADHR membrane.

**Figure 10:**
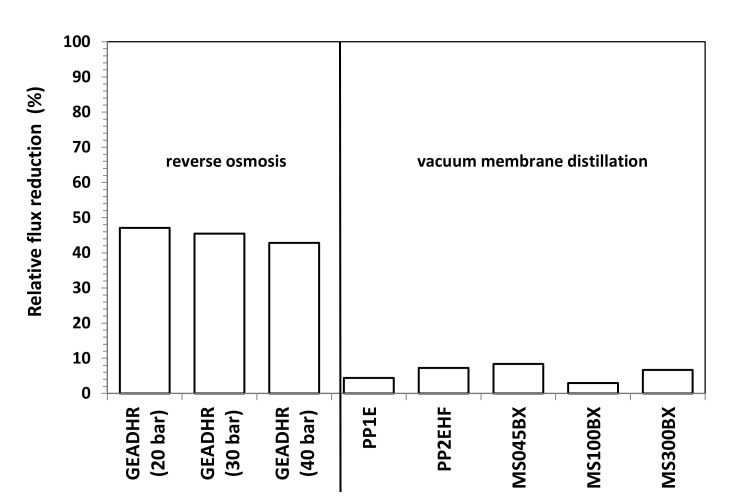
Relative flux reduction vs. operating pressure of RO and VMD membranes.

Figure 9 refers to RO tests carried at different operating pressures. Average values are considered as the permeate flux remained substantially constant with time during the various tests with fluctuations even smaller that of those observed during VMD tests, as the comparison of SD values reported in Figure 8 and Figure 9 reveals. As it can be seen from Figure 9 as well as from the RFR values reported in Figure 10 that, by feeding the RO plant with FFWW, a decay of the permeate flux much higher than that found for VMD is observed. The RFR gives an indication of the fouling occurred during the operation and FRR gives an indication of how easily the original flux can be recovered just by a simple water washing. Figure 9 shows that the flux during FFWW treatment at 20 bar is 21.2 LMH, a value slightly lower than that of the less permeable VMD membrane (PP1E, 22 LMH) (Figure 8). By increasing the pressure at 30 bar, the flux reaches a value, 32.6 LMH, similar to that of PP2HE membrane, but slightly lower than that of MS045BX membrane (34.2 LMH). A further increase in the pressure at 40 bar leads the flux to a value (44.7 LMH) much lower than that of PTFE MS100BX (51 LMH) and PTFE MS300BX membrane (57.4 LMH). From the above reported RO permeate flux and pressure data, an average permeability value of 1.09 ± 0.03 LMH/bar can be calculated. Therefore, it is easy to verify that the highest flux obtained from VMD tests can be hypothetically reached by increasing the RO pressure up to 52.7 bar—a value that, apart from the increase in pumping and energy cost, can favour polarisation and fouling phenomena. Dolar et al. [21] investigated the performance of four different RO membrane at 25 bar operating pressure with a wastewater (neutralized to a pH near 6) much less concentrated (EC = 2020 µs/cm; F^-^ = 18.7 mg/L; P_2_O_5_ = 2.4 mg/L) than that treated by us (Table 3). The authors measured permeate fluxes from 25.5 LMH to 30.5 LMH, slightly higher than those shown in Figure 9, and found a RFR of ≈21% for one of the three RO membranes and, almost surprisingly for the low concentration of the wastewater, a RFR ranging from ≈55% to ≈62% for the remaining two membranes. Unfortunately, no comparison can be made with the wastewaters considered by Karabelas et al. [20], as the authors supply neither permeate flux values nor useful indications (e.g., flow rate, membrane surface) to calculate these values.

**Figure 11:**
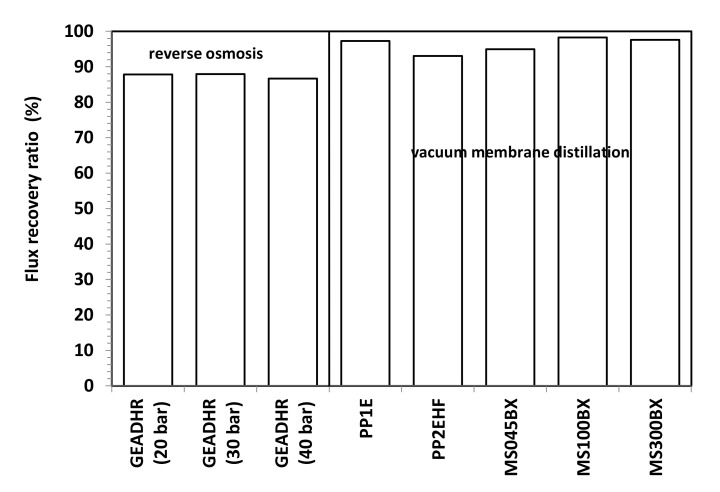
Flux recovery ratio vs. operating pressure of RO and VMD membranes.

By measuring the pure water flux H_2_O (f) again after FFWW treatment, only a partial recovery of the initial pure water flux, H_2_O (i), is obtained (Figure 9), as it can be seen better from the FRR values shown in Figure 11. The FRR is taken as a measure of membrane fouling; the lower the former, the high the latter. Therefore, from the comparison of the results shown in Figure 11 to those reported in Figure 10, it is apparent that MD has a lower fouling tendency than RO. Dolar et al. [21] had to use alkali and acid cleaning agents to remove foulants from the RO membranes and to obtain an FRR from ≈66% to ≈99%, depending on the RO membrane. The higher tendency to fouling of the RO membrane can be ascribed to its hydrophilic character.

The main physical-chemical parameters of average distillate and permeate samples collected during VMD and RO tests are reported in Table 4. As can be seen, all VMD membrane have an excellent retention (greater than 99%) for the majority of ions. Fluoride retention is the range of 96.3–97.3%, but the fluoride concentration in all types of VMD distillate is always below the Italian surface discharge limit (ISDL, Table 3). COD retention is only 88.2%, but there is no need to worry because the COD level in the wastewater is much lower that the ISDL. Similarly, pH slightly lower than 4.0, still too low for ISDL, is observed for all the VMD distillates. Thus, a neutralisation, as reported in the paper of Dolar et al. [21], has to be foreseen to increase the pH to meet the limit value imposed by the regulation for discharge.

As appears from Table 4, the retention of the RO membrane is always lower than that of the VMD membrane. As expected, for RO, an increase in the pressure improves the quality of permeate. For example, at 40 bar, the phosphate concentration reaches a value much lower than the ISDL, while the fluoride concentration decreases slowly with the rise in pressure and, at 40 bar, remains much higher (4–6 times) than the ISDL. The phosphate retention values shown in Table 4 are in reasonable agreement with those measured by Dolar et al. [21] (R%, F^−^ from 96.9 % to 96.6 % and R%, P_2_O_5_, from 95.6% to 99.1%), while fluoride retentions are lower than those reported by the authors. However, using the same three RO membranes to test artificial model wastewaters, they found lower retentions, not too different from our findings (R%, F^−^ from 74.3% to 88.8% for NaF and from 90.2% to 97.0% for CaF_2_; R%, P_2_O_5_, from 96.8% to 98.7%, depending on the type of membrane). As far as ammonium retention is concerned, Karabelas et al. [20], carrying out RO tests of a stream coming from an ammonium nitrate fertiliser production unit, measured NH_4_^+^ retentions from 92% to 97%, very close to those reported in Table 4. The authors also observed a retention improvement by neutralizing the acidic (pH = 3.5) wastewater until pH = 5.6. A more relevant positive effect of the pH on the retention was also found by treating a fluorosilicic acid stream coming from a phosphoric acid production unit. In particular, by progressively adjusting the pH of the original wastewater from 2.4 to 4.5 and to 10.2, the fluoride retentions increased from 23% to 87% and to 93%, respectively. Therefore, unsatisfactory fluoride retention reported in Table 4 can be ascribed to the acidic characteristic (pH = 3, Table 1) of the FFWW.

## 4. Conclusions

The preliminary result obtained from laboratory-scale tests demonstrates that VMD can be successfully applied to the treatment of FFWW. All tested MD membranes provide high quality distillates with a contaminant level lower than ISDL together with a too acidic pH, which, hovewer, can be adjusted by a simple neutralization step. Distillate fluxes varying from 22 to 57.4 LMH are obtained by operating at a moderate temperature (T = 40 °C) that can be easily achieved using waste heat available from production facilities. RO showed good retentions (>97%) to sulfate, phosphate, and ammonia and lower retentions (83–87%) to all the other water quality parameters. A comparison between MD and RO results reveals MD advantages not only owing to higher fluxes and better retentions, but also to a lower fouling tendency as the initial pure water flux is almost completely recovered after FFWW treatment. Moreover, keeping in mind the independence of the MD from the osmotic pressure, which, on the contrary, deeply affects RO, it is easy to think that the gap between the two processes will increase by operating with a more concentrated FFWW. This will be verified in a successive study also aimed at ascertaining process performance in long-term concentration tests, as well as at defining proper cleaning cycles, which, on the basis of both FFR and RFR ratios shown in Figure 10 and Figure 11, seems to be more necessary for RO than for MD.

## Figures and Tables

**Table 1:** Main properties of the membranes used for VMD tests.

Manufacturer/Supplier	Membrane Solutions (Ms), LLC			Membrana GmbH	
Membrane type	MS045BX	MS100BX	MS300BX	PP1E	PP2EHF
Membrane material	PTFE	PTFE	PTFE	PP	PP
Support material	PP	PP	PP	(a)	(a)
Overall thickness [µm]	170 ± 20	170 ± 20	170 ± 20	100 ± 15	170 ± 15
Pore size [µm]	0.45	1	3	-	-
Alcohol bubble point [bar]	0.7–1.0 (b)	0.4–0.5 (b)	0.3–0.4 (b)	(c)	(c)
Isopropylalcohol bubble point, T = 23 °C [bar]	-	-	-	2.1 ± 0.4	>0.76
Alcohol flux, T = 25 °C, P = 0.69 bar [mL min^−1^ cm^−2^]	35–50 (c)	60–80 (c)	180–250 (c)	-	-
Isopropylalcohol permeability, T = 23 °C [mL min^−1^ cm^−2^ bar^−1^]	-	-	-	>2.5	>8.5
References		[44]		[45]	[46]

(a) Unsupported; (b) no info on the alcohol type and temperature; (c) no info on the alcohol type.

**Table 2:** Main properties of the ADHR (GE Water&Process Technologies, Feasterville-Trevose, PA, USA) membrane used for RO tests as reported in the manufacturer datasheet. Average salt retention of a spiral wound element after 24 h operation. Individual flow rate of the original element may vary +25%/−15%. Testing conditions: 32 g/L NaCl solution; pressure of 55.16 bar; temperature of 25 °C; pH of 7.5; recovery of 7%.

Average Permeate Flux [LMH]	Average NaCl Retention [%]	Minimum NaCl Retention [%]
27.5	99.75	99.3

**Table 3:** Wastewater physical-chemical characterisation and Italian surface discharge limits (ISDLs) [47].

Feature	Unit	Level	ISDL
Electrical conductivity	µS cm^−1^	11,960	-
pH	-	3.0	5.5–9.5
Cl^−^	mg L^−1^	1986	1200
F^−^	mg L^−1^	161	6
PO_4_^3−^	mg L^−1^	3121	31
SO_4_^2−^	mg L^−1^	2290	1000
NH_4_^+^	mg L^−1^	296	15
Chemical oxygen demand	mg L^−1^	119	160

**Table 4:** Physical-chemical characterization of average MD distillate and RO permeate samples (* indicates values out of the ISDL).

Feature	Unit		Membrane							
			PP1E	PP2EHF	MS045BX	MS100BX	MS300BX	GE ADHR	GE ADHR	GE ADHR
Test			VMD	VMD	VMD	VMD	VMD	RO (20 bar)	RO (30 bar)	RO (40 bar)
pH		Value	3.7 *	3.7 *	3.6 *	3.7 *	3.8 *	3.1 *	3.2 *	3.2 *
Electrical conductivity	µS cm^−1^	Value	56.5	55.3	63.1	66.4	64.8	1758.0	1370.0	1050.0
	%	Retention	99.5	99.5	99.5	99.4	99.5	85.3	88.5	91.2
Cl^−^	mg L^−1^	Value	1.0	0.5	0.3	99.9	100.0	240.9	149.5	44.6
	%	Retention	99.9	100.0	100.0	99.0	100.0	87.9	92.5	97.8
F^−^	mg L^−1^	Value	4.4	4.2	4.8	5.4	5.9	26.2 *	24.2 *	23.2 *
	%	Retention	97.3	97.4	97.0	96.7	96.3	83.7	85.0	85.6
PO_4_^3−^	mg L^−1^	Value	0.0	0.0	0.0	0.0	0.0	70.1 *	41.6 *	13.1 *
	%	Retention	100	100	100	100	100	97.8	98.7	99.6
SO_4_^2−^	mg L^−1^	Value	0.8	0.0	0.0	0.0	0.0	38.1	21.7	6.1
	%	Retention	99.9	100	100	100	100	98.2	99.0	99.7
NH_4_^+^	mg L^−1^	Value	<2	<2	<2	<2	<2	6.4	6.4	6.4
	%	Retention	>99.3	>99.3	>99.3	>99.3	>99.3	97.7	97.7	97.7
Chemical Oxygen Demand	mg L^−1^	Value	-	-	14.0	-	15.0	-	-	15.0
	%	Retention	-	-	88.2	-	87.4	-	-	87.4
